# Association between metabolic parameters and risks of anemia and electrolyte disturbances among stages 3–5 chronic kidney disease patients in Taiwan

**DOI:** 10.1186/s12882-021-02590-w

**Published:** 2021-11-17

**Authors:** Adi Lukas Kurniawan, Ya-Lan Yang, Chien-Yeh Hsu, Rathi Paramastri, Hsiu-An Lee, Po-Yuan Ni, Mei-Yun Chin, Jane C.-J. Chao

**Affiliations:** 1 School of Nutrition and Health Sciences, College of Nutrition, Taipei Medical University, 250 Wu-Hsing Street, Taipei, 110 Taiwan; 2 Research Center for Healthcare Industry Innovation, National Taipei University of Nursing and Health Sciences, 365 Ming-De Road, Beitou District, Taipei, 112 Taiwan; 3 Diet and Nutrition Department, Shuang Ho Hospital, Taipei Medical University, 291 Jhongjheng Road, Jhongjheng District, New Taipei, 235 Taiwan; 4 Department of Information Management, National Taipei University of Nursing and Health Sciences, 365 Ming-De Road, Beitou District, Taipei, 112 Taiwan; 5 Master Program in Global Health and Development, College of Public Health, Taipei Medical University, 250 Wu-Hsing Street, Taipei, 110 Taiwan; 6 Department of Computer Science and Information Engineering, Tamkang University, 151 Yingzhuan Road, Tamsui District, New Taipei, 251 Taiwan; 7 National Health Research Institutes, 35 Keyan Road, Zhunan, Miaoli County 350 Taiwan; 8 Nutrition Research Center, Taipei Medical University Hospital, 252 Wu-Hsing Street, Taipei, 110 Taiwan

**Keywords:** Anemia, Electrolyte disturbances, Mineral disorders, Chronic kidney disease, Metabolic parameters

## Abstract

**Background:**

Anemia and electrolyte disturbances are adverse outcomes of chronic kidney disease (CKD). This study explored the association between metabolic parameters with anemia and electrolyte and mineral disorders among CKD patients in Taiwan.

**Methods:**

This cross-sectional study with a total of 2176 CKD stages 3–5 patients were collected from the Department of Nephrology at Shuang Ho Hospital, Taipei Medical University through the “Chronic Kidney Disease Common Care Network” database from December 2008 to April 2019. A multivariable-adjusted logistic regression expressed as odd ratios (OR) was performed to assess the association of metabolic parameters with anemia and electrolyte and mineral disorders.

**Results:**

Elevated diastolic blood pressure, fasting blood glucose, and glycated hemoglobin A1c (HbA1c) were associated with presence of anemia. Similarly, elevated fasting blood glucose and HbA1c were associated with hyponatremia (OR = 1.59 and 1.58, *P* for both < 0.01) and hypercalcemia (OR = 1.38 and 1.33, *P* for both < 0.05). There was no significant association in serum lipid levels with presence of anemia. However, total triglycerides, total cholesterol and low-density lipoprotein-cholesterol were only associated with presence of hypercalcemia (OR = 1.43, 1.95 and 3.08, respectively, *P* for all < 0.05).

**Conclusions:**

Elevated diastolic blood pressure, fasting blood glucose, HbA1c and blood lipids are associated with anemia or electrolyte and mineral disorders in CKD patients.

**Supplementary Information:**

The online version contains supplementary material available at 10.1186/s12882-021-02590-w.

## Background

The United States Renal Data System reported in 2016 that Taiwan has the largest incidence and prevalence of end-stage renal disease (ESRD), the advanced stage of chronic kidney disease (CKD), and has continued to rank first in the world since 2002 [1]. Thus, CKD occupied the top list of medical expenditures of national health insurance, indicating that CKD threatens the health of people in Taiwan and becomes a significant financial burden on national medical resources. Anemia is an inevitable and common consequence of patients with CKD, which can develop in the early stages of CKD and is commonly observed in more advanced stages in CKD patients [2]. A prospective cohort study in Taiwan showed that 12.0% of CKD patients in stage 3 were anemic, and the prevalence increased to 58.8 and 92.5% in CKD patients in stages 4 and 5, respectively [3]. Moreover, CKD patients with anemia are more prone to CKD progression, cardiovascular comorbidities, poor quality of life, and higher mortality [2, 4, 5].

Additionally, the kidneys play a critical role in regulating body fluid, electrolytes, and acid-base balance, and CKD can lead to metabolic acidosis, hyperkalemia, hyponatremia, hypercalcemia, and hyperphosphatemia, resulting in serious adverse outcomes such as bone mineral disorders, vascular calcification, and even mortality [6, 7]. Hyperkalemia is increasingly common with the progression of CKD and is one of the life-threatening electrolyte disorders in CKD patients, with a nearly 10-fold risk of death in stages 4 and 5 [8]. CKD patients with hyperkalemia may develop certain clinical manifestations such as muscle weakness, cardiac arrhythmias, and cardiac arrest [6]. Meanwhile, hyponatremia is the most common electrolyte abnormality in CKD patients, which is likely due to fluid overload and positively correlates with mortality and morbidity [9–11]. Similarly, hypercalcemia and hyperphosphatemia are common bone mineral disorders in CKD patients and have been associated with vascular calcification, CKD progression, cardiovascular events, and mortality [12–14]. Overall, anemia and electrolyte and mineral disorders in CKD patients have major implications for cardiovascular-related comorbidities.

Hypertension, diabetes, and lipid abnormalities are the major causes of CKD [15, 16]. Previous studies have mentioned that high fasting glucose, CKD severity, body mass index, (BMI), and serum iron are independently associated with anemia among CKD patients [17, 18]. It is also well acknowledged that the use of angiotensin-converting enzyme inhibitors, diuretic treatment, impaired bone turnover, and use of calcium-based phosphate binders have been associated with electrolyte and mineral disorders in CKD patients [19, 20]. However, the existing studies scarcely focused on whether metabolic abnormalities are associated with anemia and electrolyte disturbances in CKD patients. Therefore, our study aimed to investigate the association of metabolic parameters such as elevated blood pressure, blood glucose, triglycerides, total cholesterol, low-density lipoprotein (LDL)-cholesterol, and high-density lipoprotein (HDL)-cholesterol with anemia and electrolyte and mineral disorders in CKD patients in Taiwan.

## Methods

### Research design and patients

In this cross-sectional study, pre-ESRD patients (CKD stages 3 to 5 with proteinuria) were enrolled in the Department of Nephrology at Shuang Ho Hospital, Taipei Medical University, Taiwan from December 2008 to April 2019. The data were retrieved from the “Chronic Kidney Disease Common Care Network” that has been developed by the physician of the hospital for more than 10 years. We retrieved the patients’ data including sociodemographic information, lifestyle, medical records, anthropometry, and blood biochemical data. Before visiting the hospital for CKD treatment and participating in the pre-ESRD nutrition education program, all patients signed an informed consent allowing their data to be used without personal identification for research only. The Taipei Medical University Joint Institutional Review Board approved this study (N202001055).

The data of 4094 patients who had participated in the pre-ESRD program were retrieved from the platform. Among the 4094 CKD patients, 3885 were stages 3–5 CKD patients, and a total of 1709 patients were excluded due to a history of cardiovascular disease (CVD) (*n* = 1072), a history of chronic liver disease and cancer (*n* = 232), tuberculosis and autoimmune disease (*n* = 48), current erythropoietin treatment (*n* = 353) and a lack of estimated glomerular filtration rate (eGFR) measurement (n = 4). The reason for excluding patients with presence of CVD and other comorbidities was because the predictor variables were highly associated with CVD, thus presence of CVD may become a confounding factor in our study. Finally, 2176 CKD patients were analyzed in this study.

### Clinical and blood biochemical parameters

Anthropometric data, including body weight and height, were measured using high-accuracy ultrasonic sensors (AHS 700, Kaohsiung, Taiwan). BMI was determined using body weight (kg) divided by height squared (m^2^). Systolic and diastolic blood pressure (BP) was determined by oscillometry (OMRON HBP-9020, Taipei, Taiwan). For blood biochemical measurements, all patients were required to fast at least 8 h prior to blood tests. Blood biochemical data including hemoglobin (Hb), fasting blood glucose, albumin, triglycerides, total cholesterol, LDL-cholesterol, HDL-cholesterol, potassium (K), sodium (Na), calcium (Ca), and phosphorus (P) were determined using an auto chemical analyzer (Beckman DxC 800, California, USA). Glycated hemoglobin A1c (HbA1c) was determined by capillary electrophoresis (Sebia II, Lisses, France).

Anemia was defined as Hb < 130 g/Lfor men and < 120 g/L for women, or current iron supplementation according to the Kidney Disease Improving Global Outcomes (KDIGO) Anemia Work Group [21]. Serum calcium levels were corrected for serum albumin by using Payne’s formula: corrected calcium (C-Ca) (mmol/L = calcium (mmol/L) + 0.02 × [40 – serum albumin (g/L)]) [22]. Hyperkalemia was defined as serum potassium > 5.0 mmol/L and hyponatremia was defined as serum sodium < 135 mmol/L [6]. Hypercalcemia was defined as serum levels of corrected calcium ≥2.37 mmol/L (9.5 mg/dL), while hyperphosphatemia was defined as serum levels of phosphorus ≥1.49 mmol/L (4.6 mg/dL) based on guidelines from National Kidney Foundation [23]. Metabolic parameters were defined as: high systolic BP if ≥130 mmHg with medication, high diastolic BP if ≥85 mmHg with medication [24], high fasting blood glucose if ≥7.0 mmol/L (126 mg/dL) with treatment and high HbA1_C_ if ≥6.5% with treatment [25]. Moreover, high triglycerides if ≥2.3 mmol/L (200 mg/dL) or with treatment, high total cholesterol if ≥6.2 mmol/L (240 mg/dL) or with treatment, high LDL-cholesterol if ≥4.1 mmol/L (160 mg/dL) or with treatment and low HDL-cholesterol if < 1.04 mmol/L (40 mg/dL) or with treatment [26]. The value of eGFR was calculated using the equation of the Modification of Diet in Renal Disease study [27]. Moreover, CKD stages were classified based on eGFR values into: CKD stages 3a (45–59 mL/min/1.73 m^2^), 3b (30–44 mL/min/1.73 m^2^), 4 (15–29 mL/min/1.73 m^2^) and 5 (< 15 mL/min/1.73 m^2^).

### Other covariates

We also retrieved sociodemographic and lifestyle data such as age, sex, marital status, educational level, occupation (unemployment and professional), cigarette smoking, alcohol consumption, and physical activity from the database. Marital status was dichotomized as no (divorced, widowed, and separated) and yes (currently married). Educational level was categorized as low (high school or below) and high (above high school). Cigarette smoking and alcohol use were categorized as no, former (quit smoking or drinking), and current. Data on type of physical activity (e.g., regular walking, fast walking, jogging, dancing, gymnastics, biking or hiking), frequency (5 response options: from never to ≥7 times/week), and duration (5 response options: from never to > 90 min) were collected and retrieved from the database. Physical activity was defined as no (< 30 min/week) and yes (≥30 min/week). Current medication use, including angiotensin II receptor blockers, angiotensin-converting enzyme inhibitors, calcium channel blockers, calcium phosphate binders, iron supplements, antihypertensives, hypolipidemic agents, hypoglycemic agents, or insulin injections were also queried. Patients were categorized as no (never) and yes (≥once) for participation in the nutrition education program.

### Data analysis

Data are presented as numbers and percentages for categorical variables or mean ± standard deviation (SD) for continuous variables. For categorical variables, a chi-square test was performed to examine differences in characteristics of CKD patients with or without anemia, whereas the general linear model was used to examine differences in means for continuous variables. The association between metabolic parameters and anemia or electrolyte and mineral disorders was analyzed using a multivariable-adjusted logistic regression model, and the data are reported as odds ratio (OR) and 95% confidence interval (CI). Considering different Hb cut-off values for anemia in men and women, the association between metabolic parameters and anemia was stratified by sex. The regression models were adjusted for age, marital status, educational level, occupation, smoking status, drinking status, physical activity, drug use, nutritional education, and BMI in the association between metabolic parameters and anemia. Besides the confounders adjusted in the regression model, sex was also adjusted in the association between metabolic parameters and electrolyte and mineral disorders. Patients with ‘normal’ status in all dependent variables were selected as the reference group. A *P*-value of < 0.05 was considered statistically significant, and STATA software version 13 (STATA Corp LLC, Texas, USA) was used to perform the statistical analysis.

## Results

### Characteristics of the patients

Table 1 shows the characteristics of patients with and without anemia. Of 2176 CKD patients, 67% were anemic, and 56.1% of anemic patients were men. Patients with anemia were more likely to be older (72.1 ± 14.0 vs. 68.7 ± 13.3 years) and had higher proportions of advanced stages (stages 4 and 5: 35.5 and 29.3% vs. 20.6 and 4.6%), lower educational level (69.9% vs. 60.4%), inactive physical activity (72.6% vs. 65.4%) and drug use (61.2% vs. 56.8%) compared to those without anemia. Moreover, patients with anemia had lower BMI (25.2 ± 4.4 vs. 26.3 ± 4.2 kg/m^2^), diastolic BP (72 ± 14 vs. 75 ± 13 mmHg), albumin (39.5 ± 5.9 vs. 43.1 ± 4.5 g/L) and triglycerides (1.8 ± 1.9 vs. 1.9 ± 1.3 mmol/L) compared to those without anemia. The characteristics of CKD patients stratified by electrolyte and mineral disorders are shown in Additional file 1: Table S1. Among all CKD patients, 18.3% of patients were hyperkalemic, 10.2% of patients were hyponatremic, 12.4% of patients were hypercalcemic and 25.7% of patients were hyperphosphatemic.

**Table 1:** Characteristics of CKD patients with or without anemia^a^

Variables	Total (*n* = 2176)	Non-anemia (*n* = 719)	Anemia (*n* = 1457)	*P*^b^
Age, years	70.9 ± 13.9	68.7 ± 13.3	72.1 ± 14.0	< 0.001
20–44	113 (5.2)	39 (5.4)	74 (5.1)	
45–69	818 (37.6)	327 (45.5)	491 (33.7)	
≥ 70	1245 (57.2)	353 (49.1)	892 (61.2)	
Gender				< 0.001
Men	1284 (59.0)	467 (64.9)	817 (56.1)	
Women	892 (41.0)	252 (35.1)	640 (43.9)	
CKD stage^c^				< 0.001
Stage 3	1051 (48.3)	538 (74.8)	513 (35.2)	
Stage 4	665 (30.6)	148 (20.6)	517 (35.5)	
Stage 5	460 (21.1)	33 (4.6)	427 (29.3)	
Marital status				0.216
No	652 (30.0)	203 (28.2)	449 (30.8)	
Yes	1524 (70.0)	516 (71.8)	1008 (69.2)	
Education level				< 0.001
Low	1453 (66.8)	434 (60.4)	1019 (69.9)	
High	723 (33.2)	285 (39.6)	438 (30.1)	
Occupation				< 0.001
Unemployment	1153 (53.0)	317 (44.1)	836 (57.4)	
Professional	1023 (47.0)	402 (55.9)	621 (42.6)	
Cigarettes smoking				< 0.001
No	1620 (74.4)	520 (72.3)	1100 (75.5)	
Former	235 (10.8)	64 (8.9)	171 (11.7)	
Current	321 (14.8)	135 (18.8)	186 (12.8)	
Alcohol drinking				0.001
No	1885 (86.6)	607 (84.4)	1278 (87.7)	
Former	108 (5.0)	30 (4.2)	78 (5.4)	
Current	183 (8.4)	82 (11.4)	101 (6.9)	
Physical activity^d^				0.001
No	1527 (70.2)	470 (65.4)	1057 (72.6)	
Yes	649 (29.8)	249 (34.6)	400 (27.4)	
Drug use^e^				0.049
No	877 (40.3)	311 (43.2)	566 (38.8)	
Yes	1299 (59.7)	408 (56.8)	891 (61.2)	
Nutrition education				0.330
No	943 (43.3)	301 (41.9)	642 (44.1)	
Yes	1233 (56.7)	418 (58.1)	815 (55.9)	
BMI, kg/m^2^	25.6 ± 4.4	26.3 ± 4.2	25.2 ± 4.4	< 0.001
Systolic BP, mmHg	136 ± 20	135 ± 19	135 ± 20	0.632
Diastolic BP, mmHg	73 ± 13	75 ± 13	72 ± 14	< 0.001
Fasting blood glucose, mmol/L	6.9 ± 2.9	6.9 ± 2.8	7.0 ± 3.0	0.417
HbA1c, %	6.8 ± 2.9	6.7 ± 1.6	6.9 ± 3.5	0.217
Albumin, g/L	40.6 ± 5.7	43.1 ± 4.5	39.5 ± 5.9	< 0.001
Triglycerides, mmol/L	1.8 ± 1.7	1.9 ± 1.3	1.8 ± 1.9	< 0.001
Total cholesterol, mmol/L	4.9 ± 1.4	5.0 ± 1.3	4.9 ± 1.4	0.217
LDL-cholesterol, mmol/L	2.6 ± 0.9	2.7 ± 0.8	2.6 ± 0.9	0.079
HDL-cholesterol, mmol/L	1.2 ± 0.4	1.2 ± 0.4	1.2 ± 0.4	0.273

^a^Data are presented as number (percentage) for categorical variables and mean ± standard deviation (SD) for continuous variables

^b^The *P*-value was analyzed using the general linear model for continuous variables and chi-square test for categorical variables

^c^CKD stage 3 (30–59 mL/min/1.73 m^2^), stage 4 (15–29 mL/min/1.73 m^2^) and stage 5 (< 15 mL/min/1.73 m^2^)

^d^Physically active was defined as engaging in physical activity for ≥30 min/week

^e^Drug use: angiotensin II receptor blocker, angiotensin-converting enzyme inhibitor, calcium channel blocker or calcium phosphate binder

*Abbreviations: BMI* Body mass index, *BP* Blood pressure, *CKD* Chronic kidney disease, *HbA1c* Glycated hemoglobin A1c, *HDL* High-density lipoprotein, *LDL* Low-density lipoprotein

### Association of metabolic parameters with anemia and electrolyte and mineral disorders

The results of multivariable-adjusted logistic regression showed that CKD patients with increased values of diastolic BP (OR = 1.54, 95% CI 1.09–2.19, *P* = 0.015), fasting blood glucose (OR = 1.63, 95% CI 1.27–2.09, *P* <  0.001) or HbA1c (OR = 1.48, 95% CI 1.15–1.89, *P* = 0.002) were associated with presence of anemia compared to those with normal values (Table 2). However, no significant association was found between elevated blood lipid parameters and anemia in either sex. The results of multivariable-adjusted logistic regression also showed that CKD patients with high diastolic BP were associated with presence of hyperkalemia (OR = 1.54, 95% CI 1.08–2.21, *P* = 0.017) compared to those with normal diastolic BP. Patients with elevated fasting blood glucose were associated with presence of hyponatremia (OR = 1.59, 95% CI 1.16–2.16, *P* = 0.004), hypercalcemia (OR = 1.38, 95% CI 1.05–1.83, *P* = 0.025) or hyperphosphatemia (OR = 1.32, 95% CI 1.06–1.65, *P* = 0.014). Additionally, CKD patients with elevated HbA1c were significantly associated with presence of hyperkalemia (OR = 1.26, 95% CI 1.00–1.59, *P* = 0.049), hyponatremia (OR = 1.58, 95% CI 1.16–2.16, *P* = 0.004) or hypercalcemia (OR = 1.33, 95% CI 1.00–1.76, *P* = 0.049). Meanwhile, CKD patients with high levels of triglycerides (OR = 1.43, 95% CI 1.03–1.99, *P* = 0.034), total cholesterol (OR = 1.95, 95% CI 1.28–2.96, *P* = 0.002) and LDL-cholesterol (OR = 3.08, 95% CI 1.77–5.34, *P* <  0.001) were significantly associated with hypercalcemia only. Moreover, anemic CKD patients had a significant association with presence of hyperkalemia (OR = 3.13, 95% CI 2.32–4.22, *P* < 0.001), hyponatremia (OR = 2.28, 95% CI 1.54–3.38, *P* < 0.001) and hyperphosphatemia (OR = 3.91, 95% CI 2.93–5.20, *P* < 0.001) (Table 3).

**Table 2:** Adjusted odds ratios (OR) and 95% confidence intervals (CI) for anemia, electrolyte and mineral disorders associated with metabolic parameters^a^

Metabolic parameters	Anemia OR (95% CI)	*P*	Hyperkalemia OR (95% CI)	*P*	Hyponatremia OR (95% CI)	*P*	Hypercalcemia OR (95% CI)	*P*	Hyperphosphatemia OR (95% CI)	*P*
Systolic BP										
Normal	1.00		1.00		1.00		1.00		1.00	
High	1.29 (0.95–1.78)	0.10	1.17 (0.77–1.78)	0.45	0.87 (0.53–1.44)	0.60	1.03 (0.63–1.67)	0.91	0.99 (0.66–1.47)	0.95
Diastolic BP										
Normal	1.00		1.00		1.00		1.00		1.00	
High	1.32 (1.01–1.73)	0.041	1.54 (1.08–2.21)	0.017	0.99 (0.64–1.52)	0.95	1.05 (0.70–1.58)	0.82	1.16 (0.83–1.62)	0.39
Fasting blood glucose										
Normal	1.00		1.00		1.00		1.00		1.00	
High	1.45 (1.20–1.76)	< 0.001	1.24 (0.98–1.57)	0.066	1.59 (1.16–2.16)	0.004	1.38 (1.05–1.83)	0.025	1.32 (1.06–1.65)	0.014
HbA1c										
Normal	1.00		1.00		1.00		1.00		1.00	
High	1.45 (1.19–1.76)	< 0.001	1.26 (1.00–1.59)	0.049	1.58 (1.16–2.16)	0.004	1.33 (1.00–1.76)	0.049	1.20 (0.96–1.51)	0.10
Triglycerides										
Normal	1.00		1.00		1.00		1.00		1.00	
High	0.84 (0.66–1.06)	0.15	1.00 (0.75–1.33)	1.00	1.07 (0.72–1.59)	0.72	1.43 (1.03–1.99)	0.034	1.01 (0.78–1.33)	0.92
Total cholesterol										
Normal	1.00		1.00		1.00		1.00		1.00	
High	0.85 (0.61–1.19)	0.35	1.03 (0.70–1.52)	0.82	1.35 (0.82–2.22)	0.24	1.95 (1.28–2.96)	0.002	1.24 (0.88–1.76)	0.22
LDL-cholesterol										
Normal	1.00		1.00		1.00		1.00		1.00	
High	1.53 (0.92–2.57)	0.10	0.78 (0.42–1.47)	0.45	1.56 (0.75–3.23)	0.24	3.08 (1.77–5.34)	< 0.001	1.38 (0.84–2.27)	0.21
HDL-cholesterol										
Normal	1.00		1.00		1.00		1.00		1.00	
Low	1.26 (0.92–1.72)	0.15	0.93 (0.62–1.39)	0.49	0.88 (0.49–1.56)	0.66	0.77 (0.47–1.24)	0.28	1.40 (0.96–2.02)	0.07

^a^Adjusted for age, gender, marital status, education level, occupation, smoking status, drinking status, physical activity, drug use, nutrition education and body mass index

*Abbreviations: BP* Blood pressure, *HbA1c* Glycated hemoglobin A1c, *HDL* High-density lipoprotein, *LDL* Low-density lipoprotein

**Table 3:** Adjusted odds ratios (OR) and 95% confidence intervals (CI) for electrolyte and mineral disorders associated with anemia^a^

Dependent variables	Non-anemia (Ref)	Anemia OR (95% CI)	*P*
Hyperkalemia	1.00	3.13 (2.32–4.22)	< 0.001
Hyponatremia	1.00	2.28 (1.54–3.38)	< 0.001
Hypercalcemia	1.00	1.02 (0.75–1.37)	0.92
Hyperphosphatemia	1.00	3.91 (2.93–5.20)	< 0.001

^a^Adjusted for age, gender, marital status, education level, occupation, smoking status, drinking status, physical activity, drug use, nutrition education and body mass index

## Discussion

In our study, anemic CKD patients were more likely to be older and male, and 67% of the subjects in stages 3–5 had anemia. Additionally, high fasting blood glucose and HbA1c levels were associated with presence of anemia and electrolyte and mineral imbalance including hyponatremia, hypercalcemia, and hyperphosphatemia. Similar results were also found in Japanese CKD patients, in whom the prevalence of anemia in stages 4 and 5 was 40.1 and 60.3%, respectively [28]. Age, gender, and presence of complications could be the factors affecting the development and/or severity of anemia in CKD patients [28, 29]. A previous study also showed that CKD patients with a diastolic BP between 80 and 89 mmHg had a lower risk of anemia (OR = 0.38, 95% CI 0.16–0.92) compared to those with a diastolic BP between 60 and 79 mmHg, but systolic BP was not significantly associated with anemia risk [30]. The prevalence of anemia was higher in uncontrolled hypertensives than in well-controlled hypertensives, suggesting that hypertension is independently associated with increased anemia risk [31]. Moreover, presence of anemia was independently associated with renal events even in overall hypertensive patients with well-controlled blood pressure [32]. The progression of anemia in CKD patients may be related to the left ventricular hypertrophy that exists in persistent renal failure in combination with decreased Hb levels and increased BP [33].

Similar findings were reported in diabetic patients that the incidence of anemia (33.5% vs. 27.9%) was increased in diabetic patients with uncontrolled HbA1c levels (> 7.5%) compared to those with controlled HbA1c levels (≤7.5%) [34]. Chronic hyperglycemia could lead to a cellular hypoxic state in the renal interstitium, which contributes to impaired production of erythropoietin in renal peritubular fibroblasts [35, 36]. Low erythropoietin levels are a major cause of early anemia in patients with impaired glucose homeostasis [36]. Moreover, anemia per se could be a risk factor for hyperglycemia. Elevated HbA1c levels were associated with decreased serum transferrin saturation, ferritin and mean corpuscular hemoglobin levels in Japanese diabetic women in late pregnancy, and decreased anemia parameters (24–35 weeks of gestation) occurred before elevated HbA1c levels (32–35 weeks of gestation) [37], indicating that anemia might be one of the risk factors for hyperglycemia. Thus, these results suggest that anemia could be a risk factor and consequence of hyperglycemia [35–37].

Electrolyte and mineral imbalances are common in patients with impaired renal function because the kidney plays an important role in regulating body fluid, electrolytes, acid-base balance, and iron metabolism [6]. Additionally, anemia in patients with impaired renal function may be associated with disturbance in the metabolism of calcium, phosphate, and hydrogen ions [38]. An increased risk of developing hyperkalemia has also been found in hypertensive patients on antihypertensive therapy [39]. Likewise, the present study showed that CKD patients with high diastolic BP were associated with presence of hyperkalemia. Imbalance of electrolytes potassium, sodium, and calcium were observed more frequently in patients with type 2 diabetes (> 70%) than in patients with hypoglycemia or normal fasting blood glucose, and changes in electrolyte distribution could be due to osmotic fluid shift by hyperglycemia or loss of electrolytes by osmotic diuresis [40]. Our study showed that high HbA1c was significantly associated with presence of hyperkalemia in CKD patients (*P* = 0.049). Similarly, a study in Benin found that mean serum potassium levels were significantly higher in diabetic patients compared to non-diabetic controls of the same age and sex [41].

Our results showed that high fasting blood glucose and high HbA1c were associated with presence of hyponatremia in CKD patients. Similarly, a previous study found that hyperglycemia in healthy adults with acute insulin deficiency rapidly decreased serum sodium levels, and hyponatremia was reversed by normoglycemia [42]. The plausible mechanism is that hyperglycemia could increase serum osmolality, cause efflux of water from cells, and lead to further hyponatremia due to dilution [42]. Additionally, our results showed that high fasting blood glucose and HbA1c were associated with presence of hypercalcemia and hyperphosphatemia in CKD patients. Consistent with our findings, serum calcium levels were positively associated with fasting blood glucose and insulin resistance, but negatively associated with pancreatic β-cell function in healthy adults [43], suggesting that abnormal regulation of calcium homeostasis may be related to impaired β-cell function and elevated glucose levels. According to the results of previous and current studies, the relationship between hyperglycemia and hypercalcemia could be cyclic. Hyperphosphatemia frequently occurred in late-stage CKD patients because the excretory capacity of phosphate by the kidney is exhausted [44, 45]. Moreover, hyperphosphatemia has been associated with vascular calcification and abnormal bone mineralization and turnover [46], which was frequently and positively associated with CVD morbidity and mortality in CKD or diabetic patients [47]. Combined hyperphosphatemia with hyperglycemia promoted vascular calcification in human aortic smooth muscle cells compared with hyperphosphatemia or hyperglycemia alone [47]. Similar to the previous studies [48, 49], we also found a strong association between hyperphosphatemia and presence of anemia. High serum phosphorus may lead to increases in the production of uremic toxins as higher polyamines and the secretion of parathyroid hormone which has been shown to inhibit erythropoiesis [48].

The present study showed an association between blood lipids and hypercalcemia in CKD patients. Similarly, previous studies also found a positive association between serum calcium and triglycerides, total cholesterol or HDL-cholesterol [50, 51]. Elevated calcium levels contributed to a decrease in hepatic cholesterol catabolism via a reduction in 7α-hydroxylase, and to an increase in de novo lipid synthesis via an increase in sterol regulatory element-binding protein-1c, which may serve as a plausible mechanism for this association [50]. However, in the present study, no significant relationship was found between lipid profile and anemia. Previous studies also found no significant association between Hb levels and serum lipid concentrations [52, 53], and the results have been inconsistent [54]. Therefore, it should be further investigated whether and how iron status affects serum lipids in CKD patients.

This study had several limitations. First, this study was a cross-sectional design, making it difficult to draw a causal conclusion using our model. A longitudinal study is needed to clarify the relationship. Second, we did not collect data on patients’ dietary habits in relation to mineral or iron sources. Third, the definition of anemia in the present study lacks other parameters such as serum iron, ferritin, transferrin saturation, and total iron-binding capacity. For clinical diagnosis of anemia in CKD patients, measurement of hemoglobin along with body iron status is recommended. The strength of our study is that it includes a large sample population of CKD patients, which provides better evidence for general public interpretation.

## Conclusions

In summary, fasting blood glucose and HbA1c are associated with presence of anemia, hyperkalemia, hyponatremia, hypercalcemia, and hyperphosphatemia in patients with stage 3–5 CKD. Moreover, the results of the present study suggest that there is a discordant association of blood pressure or serum lipid concentrations with anemia or electrolyte and mineral imbalances. A longitudinal study with prospective measurements is needed to further investigate this association.

## Supplementary Information

**Additional file 1: Table S1**. Characteristics of CKD patients stratified by electrolyte and mineral disorders.

## Data Availability

The data that support the findings of this study are available from the Department of Nephrology at Shuang Ho Hospital, Taipei Medical University, but restricted for research use only. The data are not publicly available. Data are available from the authors upon reasonable request and with permission of Shuang Ho Hospital, Taipei Medical University.

## Abbreviations

BMI: Body mass index
BP: Blood pressure
Ca: Calcium
C-Ca: Corrected calcium
CKD: Chronic kidney disease
eGFR: Estimated glomerular filtration rate
ESRD: End stage renal disease
Hb: Hemoglobin
HbA1c: Glycated hemoglobin A1c
HDL: High-density lipoprotein
K: Potassium
LDL: Low-density lipoprotein
Na: Sodium
P: Phosphorus
