# Supplementary material for: Association between metabolic parameters and risks of anemia and electrolyte disturbances among stages 3–5 chronic kidney disease patients in Taiwan
Source: BMC Nephrol. 2021 Nov 17;22:385. doi: 10.1186/s12882-021-02590-w (PMC8600925; doi:10.1186/s12882-021-02590-w)
Supplement: Supplementary file 1 — Additional file 1: Table S1. Characteristics of CKD patients stratified by electrolyte and mineral disorders. [file 12882_2021_2590_MOESM1_ESM.docx]

**Association between metabolic parameters and risks of anemia and electrolyte disturbances among stages 3-5 chronic kidney disease patients in Taiwan**

Adi Lukas Kurniawan^1,2^, Ya-Lan Yang^3^, Chien-Yeh Hsu^4,5^, Rathi Paramastri^1^, Hsiu-An Lee^6,7^, Po-Yuan Ni^1^, Mei-Yun Chin^3^* and Jane C.-J. Chao^1,5,8^*

**Table S1** Characteristics of CKD patients stratified by electrolyte and mineral disorders^a^

| Variables | Hyperkalemia ^b^ | | | Hyponatremia ^c^ | | | Hypercalcemia ^d^ | | | Hyperphosphatemia ^e^ | | |
| --- | --- | --- | --- | --- | --- | --- | --- | --- | --- | --- | --- | --- |
|  | No | Yes | *P*^f^ | No | Yes | *P*^f^ | No | Yes | *P*^f^ | No | Yes | *P*^f^ |
| Age, years |  |  | NS |  |  | NS |  |  | **0.003** |  |  | **<0.001** |
| 20–44 | 90 (5.2) | 20 (5.2) |  | 99 (5.3) | 11 (5.2) |  | 101 (5.8) | 6 (2.4) |  | 55 (3.7) | 50 (9.9) |  |
| 45–69 | 625 (36.3) | 156 (40.5) |  | 696 (37.0) | 77 (36.1) |  | 660 (38.1) | 76 (30.9) |  | 508 (34.7) | 229 (45.2) |  |
| ≥70 | 1006 (58.5) | 209 (54.3) |  | 1087 (57.7) | 125 (58.7) |  | 974 (56.1) | 164 (66.7) |  | 902 (61.6) | 227 (44.9) |  |
| Gender |  |  | NS |  |  | NS |  |  | **<0.001** |  |  | **<0.001** |
| Men | 1001 (58.2) | 241 (62.6) |  | 1121 (59.6) | 115 (54.0) |  | 1052 (60.6) | 113 (45.9) |  | 925 (63.1) | 242 (47.8) |  |
| Women | 720 (41.8) | 144 (37.4) |  | 761 (40.4) | 98 (46.0) |  | 683 (39.4) | 133 (54.1) |  | 540 (36.9) | 264 (52.2) |  |
| CKD stage^g^ |  |  | **<0.001** |  |  | **<0.001** |  |  | NS |  |  | **<0.001** |
| Stage 3 | 883 (51.3) | 115 (29.9) |  | 926 (49.2) | 66 (31.0) |  | 820 (47.3) | 116 (47.2) |  | 844 (57.6) | 86 (17.0) |  |
| Stage 4 | 496 (28.8) | 157 (40.8) |  | 582 (30.9) | 68 (31.9) |  | 533 (30.7) | 83 (33.7) |  | 466 (31.8) | 145 (28.7) |  |
| Stage 5 | 342 (19.9) | 113 (29.3) |  | 374 (19.9) | 79 (37.1) |  | 382 (22.0) | 47 (19.1) |  | 155 (10.6) | 275 (54.3) |  |
| Marital status |  |  | NS |  |  | NS |  |  | NS |  |  | NS |
| No | 532 (30.9) | 105 (27.3) |  | 565 (30.0) | 69 (32.4) |  | 515 (29.7) | 77 (31.3) |  | 430 (29.4) | 156 (30.9) |  |
| Yes | 1189 (69.1) | 280 (72.7) |  | 1317 (70.0) | 144 (67.6) |  | 1220 (70.3) | 169 (68.7) |  | 1035 (70.6) | 350 (69.2) |  |
| Education level |  |  | NS |  |  | **0.013** |  |  | **0.011** |  |  | NS |
| Low | 1155 (67.1) | 249 (64.7) |  | 1237 (65.7) | 158 (74.2) |  | 1128 (65.0) | 180 (73.2) |  | 950 (64.8) | 348 (68.8) |  |
| High | 566 (32.9) | 136 (35.3) |  | 645 (34.3) | 55 (25.8) |  | 607 (35.0) | 66 (26.8) |  | 515 (35.2) | 158 (31.2) |  |
| Cigarettes smoking |  |  | NS |  |  | NS |  |  | NS |  |  | NS |
| No | 1296 (75.3) | 273 (70.9) |  | 1398 (74.3) | 162 (76.1) |  | 1283 (74.0) | 192 (78.1) |  | 1095 (74.7) | 367 (72.5) |  |
| Past | 180 (10.5) | 45 (11.7) |  | 202 (10.7) | 22 (10.3) |  | 186 (10.7) | 22 (8.9) |  | 161 (11.0) | 50 (9.9) |  |
| Current | 245 (14.2) | 67 (17.4) |  | 282 (15.0) | 29 (13.6) |  | 266 (15.3) | 32 (13.0) |  | 209 (14.3) | 89 (17.6) |  |
| Alcohol drinking |  |  | NS |  |  | NS |  |  | **0.02** |  |  | NS |
| No | 1496 (86.9) | 328 (85.2) |  | 1622 (86.2) | 194 (91.1) |  | 1487 (85.7) | 226 (91.9) |  | 1258 (85.9) | 442 (87.4) |  |
| Past | 80 (4.7) | 23 (6.0) |  | 166 (8.8) | 9 (4.2) |  | 88 (5.1) | 9 (3.7) |  | 69 (4.7) | 30 (5.9) |  |
| Current | 145 (8.4) | 34 (8.8) |  | 94 (5.0) | 10 (4.7) |  | 160 (9.2) | 11 (4.4) |  | 138 (9.4) | 34 (6.7) |  |
| Physical activity^h^ |  |  | NS |  |  | NS |  |  | NS |  |  | **<0.001** |
| No | 1215 (70.6) | 265 (68.8) |  | 1313 (69.8) | 161 (75.6) |  | 1203 (69.3) | 176 (71.5) |  | 984 (67.2) | 389 (76.9) |  |
| Yes | 506 (29.4) | 120 (31.2) |  | 569 (30.2) | 52 (24.4) |  | 532 (30.7) | 70 (28.5) |  | 481 (32.8) | 117 (23.1) |  |
| Drug use^i^ |  |  | NS |  |  | NS |  |  | NS |  |  | 0.011 |
| No | 680 (39.5) | 152 (39.5) |  | 743 (39.5) | 85 (39.9) |  | 684 (39.4) | 92 (37.4) |  | 597 (40.7) | 174 (34.4) |  |
| Yes | 1041 (60.5) | 233 (60.5) |  | 1139 (60.5) | 128 (60.1) |  | 1051 (60.6) | 154 (62.6) |  | 868 (59.3) | 332 (65.6) |  |
| Nutrition education |  |  | NS |  |  | **0.001** |  |  | **0.039** |  |  | NS |
| No | 746 (43.4) | 149 (38.7) |  | 1107 (58.8) | 99 (46.5) |  | 1044 (60.2) | 131 (53.2) |  | 876 (59.8) | 290 (57.3) |  |
| Yes | 975 (56.7) | 236 (61.3) |  | 775 (41.2) | 114 (53.5) |  | 691 (39.8) | 115 (46.8) |  | 589 (40.2) | 216 (42.7) |  |
| BMI, kg/m^2^ | 25.6 ± 4.4 | 25.4 ± 4.4 | NS | 25.7 ± 4.4 | 24.2 ± 4.3 | **<0.001** | 25.6 ± 4.4 | 25.7 ± 4.4 | NS | 25.6 ± 4.2 | 25.5 ± 4.8 | NS |
| Systolic BP, mmHg | 135 ± 20 | 137 ± 20 | NS | 136 ± 20 | 133 ± 19 | **0.028** | 136 ± 19 | 136 ± 20 | NS | 135 ± 19 | 138 ± 21 | **0.004** |
| Diastolic BP, mmHg | 73 ± 13 | 73 ± 13 | NS | 73 ± 13 | 71 ± 14 | **0.008** | 73 ± 14 | 72 ± 13 | NS | 73 ± 13 | 74 ± 15 | NS |
| Fasting blood glucose,  mmol/L | 6.9 ± 3.0 | 7.1 ± 2.7 | NS | 6.8 ± 2.7 | 7.7 ± 4.2 | **0.031** | 6.8 ± 2.7 | 7.6 ± 4.0 | **0.004** | 6.9 ± 3.4 | 7.0 ± 2.7 | NS |
| HbA1c, % | 6.8 ± 1.9 | 7.0 ± 5.6 | NS | 6.8 ± 3.0 | 7.3 ± 2.1 | **<0.001** | 6.7 ± 3.1 | 7.3 ± 2.0 | **<0.001** | 6.8 ± 3.4 | 6.7 ± 1.4 | NS |
| Triglycerides, mmol/L | 1.8 ± 1.8 | 1.8 ± 1.3 | NS | 1.8 ± 1.7 | 1.8 ± 1.4 | NS | 1.8 ± 1.7 | 1.9 ± 1.4 | **0.01** | 1.8 ± 1.7 | 1.9 ± 1.3 | **0.006** |
| Total cholesterol, mmol/L | 4.9 ± 1.4 | 4.8 ± 1.3 | **0.048** | 4.9 ± 1.3 | 4.9 ± 1.6 | NS | 4.9 ± 1.3 | 5.2 ± 1.6 | **0.005** | 4.9 ± 1.3 | 5.0 ± 1.5 | NS |
| LDL-cholesterol, mmol/L | 2.6 ± 0.9 | 2.5 ± 0.8 | **0.043** | 2.6 ± 0.9 | 2.5 ± 1.0 | NS | 2.6 ± 0.8 | 2.8 ± 1.2 | NS | 2.6 ± 0.9 | 2.6 ± 1.0 | NS |
| HDL-cholesterol, mmol/L | 1.2 ± 0.4 | 1.2 ± 0.4 | NS | 1.2 ± 0.4 | 1.2 ± 0.5 | NS | 1.2 ± 0.4 | 1.3 ± 0.5 | **0.01** | 1.2 ± 0.4 | 1.2 ± 0.4 | NS |

^a^Data are presented as number (percentage) for categorical variables and mean ± standard deviation (SD) for continuous variables.

^b^Defined as potassium >5.0 mmol/L.

^c^Defined as sodium <135 mmol/L.

^d^Defined as corrected calcium >2.37 mmol/L.

^e^Defined as phosphorus >1.49 mmol/L.

^f^The *P*-value was analyzed using the general linear model for continuous variables and chi-square test for categorical variables.

^g^CKD stage 3 (30-59 mL/min/1.73 m^2^), stage 4 (15-29 mL/min/1.73 m^2^) and stage 5 (<15 mL/min/1.73 m^2^).

^h^Physically active was defined as engaging in physical activity for ≥30 minutes/week.

^i^Drug use: angiotensin II receptor blocker, angiotensin-converting enzyme inhibitor, calcium channel blocker or calcium phosphate binder.

*Abbreviations:* *BMI* body mass index, *BP* blood pressure, *CKD* chronic kidney disease, *HbA1c* glycated hemoglobin A1c, *HDL* high-density lipoprotein, *LDL* low-density lipoprotein, *NS* not significant.
